# Effects of E-Cigarette (e-cig) Aerosols on Mutagenesis in Selected Organs in a C57 *lacI* (BigBlue^TM^) Mouse Model

**DOI:** 10.3390/ijerph21121693

**Published:** 2024-12-19

**Authors:** Dweet Chhaya, Merna Gress, Amna Raja, Wieslawa Kosinska, Terry Gordon, Judith Zelikoff, Joseph B. Guttenplan

**Affiliations:** 1 Division of Environmental Medicine, Grossman School of Medicine, New York University, New York, NY 10016, USA; dgc9748@nyu.edu (D.C.); ar5240@nyu.edu (A.R.); terry.gordon@nyulangone.org (T.G.); judith.zelikoff@nyulangone.org (J.Z.); 2 Department of Periodontics and Oral Medicine, School of Dentistry, University of Michigan, Ann Arbor, MI 48104, USA; mg7231@nyu.edu; 3 Department of Molecular Pathobiology, New York University College of Dentistry, New York, NY 10010, USA; wk1@nyu.edu

**Keywords:** e-cigarette, aerosol, ENDS, mutagenesis, mouse, organs

## Abstract

The objective of this study is to investigate the potential mutagenic effects of the exposure of mice to aerosols produced from the component liquids of an electronic nicotine delivery system (ENDS). The use of electronic cigarettes (e-cigs) and ENDSs has increased tremendously over the past two decades. From what we know to date, ENDSs contain much lower levels of known carcinogens than tobacco smoke. While conventional tobacco smoke is a well-established mutagen, little is known about the mutagenicity of ENDS aerosols. Here, we report the mutagenic effects of a 3-month whole body exposure of C57 *lacI* mice (BigBlue^TM^) to filtered air (AIR) or ENDS aerosols in several tissues. Aerosols were generated from a 50/50 vegetable glycerin (VG)/propylene glycol (PG) mixture with and without nicotine. The results revealed that in the lung, bladder urothelial tissue, and tongue, mutagenesis was significantly greater in the VG/PG/nicotine group than in the AIR group. In all organs except the bladder, mutagenesis in the VG/PG only group was similar to those exposed to AIR. In the bladder, mutagenesis in the VG/PG group was elevated compared to that in the AIR group. In the liver, mutagenesis was modestly elevated in the VG/PG/nicotine group, but the elevation failed to reach statistical significance. Overall, there were no consistent differences in mutagenesis between the sexes. The results of this study suggest that exposure to e-cig aerosols containing nicotine represents a risk factor for carcinogenesis in several organ systems, and exposure to VG/PG alone may be a risk factor for bladder cancer.

## 1. Introduction

E-cigs were first introduced about two decades ago, and they and other electronic nicotine delivery systems (ENDSs) have rapidly become popular in the US and many other countries [1,2]. A wide variety of ENDSs (e.g., E-cigs, e-cigs, vape pens, mods, and tank systems) have evolved [3]. Particularly concerning is their popularity among younger individuals [2,4]. While aerosols from ENDSs contain lower levels of known carcinogens than smoke from conventional tobacco cigarettes, they contain relatively high levels of small aldehydes [5]. In addition, nicotine in ENDS aerosols can be converted to the carcinogenic N-nitrosamines, N-nitrosonornicotine (NNN), 4-(methylnitrosamino)-1-(3-pyridyl)-1-butanol (NNK), 4-(methylnitrosamino)-1-(3-pyridyl)-1-butanol (NNAL), and perhaps others in vivo [6,7].

Little is known about the long-term effects of continued usage of ENDSs, particularly their potential carcinogenicity. It is still too soon for epidemiological studies to establish whether there are associations between the long-term usage of ENDSs and cancer in users. Possible dangers of long-term usage have been pointed out [8]. There are in vitro studies reporting that e-cigarette liquid or vapor can induce DNA damage and oxidative stress in different types of oral cells [9,10,11]. The exposure of mice to high-voltage ENDS aerosol led to increased levels of the oxidative stress marker 8-oxodeoxyguanosine in the lungs [12]. There are also reports that the exposure of mice to e-cig aerosols can lead to DNA adducts in several organs and to tumors in the lungs [13,14].

Thus, we hypothesized that ENDS exposure can lead to mutagenesis. Here, we investigated the potential mutagenic effects of the exposure of mice to aerosols produced from the component liquids of an electronic nicotine delivery system (ENDS) and report that 3-month exposure of *lacI* mice (BigBlue^TM^) to E-cig aerosols leads to mutagenesis in the lung, urinary bladder, tongue, and possibly the liver.

## 2. Materials and Methods

### 2.1. Animals and Housing

Two-month-old Big Blue Transgenic mice were kindly provided by Mr. Robert Young (MilliporeSigma, BioReliance Toxicology Testing Services, Rockville, MD, USA, now Gentronix, Alderly Park, Macclesfield, UK). Unfortunately, due to COVID 19-related delays, the mice were not used until 12 months of age. The animals were housed in an Innovive IVC Rodent caging system at a temperature range between 19 and 25 °C (30–70% humidity) and provided standard rodent feed and water *ad libitum*, except during exposure. Wood chips were used as the housing cage bedding and cages were changed once a week. Animals were monitored twice daily for mortality and once daily for clinical signs of distress during the exposure period. The animals were also observed for clinical signs of distress during the 4 h exposure period. Two female mice in the VG/PG group were found dead during the treatment period.

### 2.2. Exposure

The animals were divided randomly into three exposure groups of 12 or 13 mice each (6M + 6 or 7F) and treated via whole-body exposure to either (1) HEPA-filtered air (Air control); (2) 50:50 ratio of vegetable glycerin/propylene glycol (VG/PG); or (3) VG/PG + 24 mg/mL nicotine (VG/PG + nicotine) for 4 h/day, 5 days/week, for 90 days in whole-body inhalation exposure chambers (Hazelton 1000) at the NYU School of Medicine’s Inhalation Exposure Core.

### 2.3. E-cig Aerosol Generation

E-cig aerosols were generated using a 3 port Chrontrol-controlled e-cig aerosol generator (~e-Aerosols, Central Valley, NY, USA). E-cig cartomizer and cartridges (Kangertech, Shenzhen, Guangdong, China) were attached to the aerosol generator operating at 4.0–4.7 V and 75 L/min air flow rate with a puff regimen of 4 sec puffs every 30 s (55 mL puff volume). The flow rate was calibrated each day using a Bios Dry Cal DC Lite flow calibrator (Bios International (bought out by Mesa Labs, Bozeman, MT, USA). The chamber flow rate was 75 L/min and the chamber static pressure was held at approximately 0.1 inches negative water pressure. The chamber levels of fine particulate matter (PM_2.5_) concentrations were determined gravimetrically using Whatman Cytiva PTFE (Polypropylene backed) membrane filters with 1.0 µm pore size and 47 mm diameter (Cat no. 7590-004, Lot no. 00715540) and were also monitored at regular intervals using a Thermo Fisher PDR 1500 (Thermo Fisher, Pittsburg, PA, USA). The mean gravimetric chamber PM_2.5_ concentration over the 3-month exposure period was 111.1 mg/m^3^ for PG/VG and 133.4 mg/m^3^ for PG/VG + nicotine. After euthanasia with CO_2_ followed by cervical dislocation, the tissues of interest were collected and transferred to a −80° freezer until used.

### 2.4. Mutagenesis

*lacI* (Big Blue^TM^) rodents contain a lambda shuttle vector that includes the bacterial *lacI* locus and the *cII* gene, which is the target for mutagenesis studies. The assay detects mutations at the *cII* locus and possibly the regulator *cI* locus [15,16]. The *cII* protein is a positive regulator of gene transcription that controls the decision between lytic or lysogenic development pathways in phage-infected cells. In appropriate *E. coli* (*E. coli* 1250) host cells under specified conditions (25 °C), only mutants give rise to phage plaques, whereas at 37 °C, all infected cells give rise to plaques, providing a phage titer. The ratio of mutant to non-mutant plaques is the measure of mutagenesis, known as the mutant fraction (MF) [15,16].

DNA isolation from tongue and bladder urothelial tissue was performed as previously described using an ammonium acetate precipitation method [17]. Several bladders did not yield sufficient DNA to perform the mutagenesis assays, but sample sizes were still sufficient for statistical evaluation. DNA from liver and lung tissue was homogenized in a Dounce homogenizer, and nuclei were separated by centrifugation. The nuclei were digested with protease K, and RNase A was added before layering this mixture onto 0.45 µm pore-size membranes and dialyzing overnight [17].

After DNA isolation, phage packaging was carried out using a homemade packaging extract prepared from bacterial strains supplied by Dr. Peter Glazer (Yale University School of Medicine, New Haven, CT, USA). The phage suspension was mixed with the appropriate host strain of *E. coli* (1250) and assayed for plaque-forming units (pfu) and mutants. At least three packaging reactions were carried out for each DNA sample.

### 2.5. Result Analysis

An unpaired Student’s *t*-test was utilized to examine the significance of the differences between groups. A *p* value < 0.1 was considered statistically significant. Excel software in Microsoft Office 365 was used for statistical calculations.

## 3. Results

Mutagenesis was measured in four body tissues (lung, tongue, bladder, and liver) following three months of exposure to either filtered air, PG/VG, or PG/VG + nicotine. Two of these tissues (lung and tongue) had contact with e-cig aerosol or its condensate. One (bladder) had exposure to unmetabolized e-cig aerosol components and its excreted metabolites in urine, and one (liver) had indirect exposure. Table 1 summarizes the mutagenesis data observed in this study.

**Figure 1:**
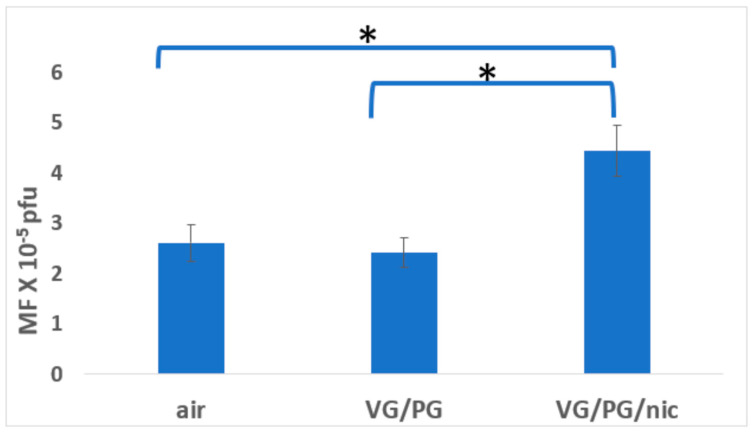
Effect of 3-month whole-body exposure of C57 *lacI* mice (Big Blue) to e-cig aerosols or filtered air on mutagenesis in lung. E-cig aerosol without nicotine (VG/PG); E-cig aerosol with nicotine, (VG/PG/nic), pfu (plaque-forming units). All groups consist of females + males. n = 13 for air, 10 for VG/PG, and 12 for VG/PG/nic groups. *, *p* ≤ 0.01 vs. filtered air in one-tailed *t*-test; error bars, mean ± SEM. MF, mutant fraction.

**Figure 2:**
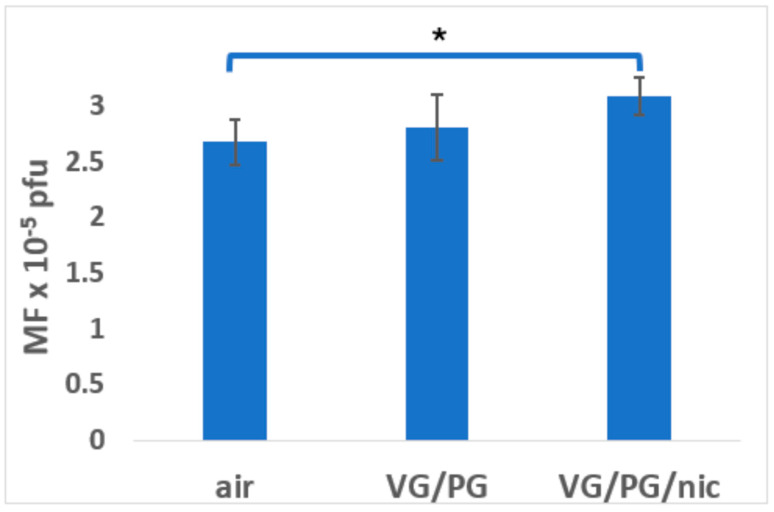
Effect of 3-month whole-body exposure of C57 *lacI* mice (Big Blue) to e-cig aerosols or filtered air on mutagenesis in tongue. E-cig aerosol without nicotine (VG/PG); E-cig aerosol with nicotine, (VG/PG/nic), pfu (plaque-forming units). All groups consist of females + males. n = 13 for air, 10 for VG/PG, and 12 for VG/PG/nic groups. *, *p* ≤ 0.05 vs. filtered air group in one-tailed *t*-test; error bars, mean ± SEM.

**Figure 3:**
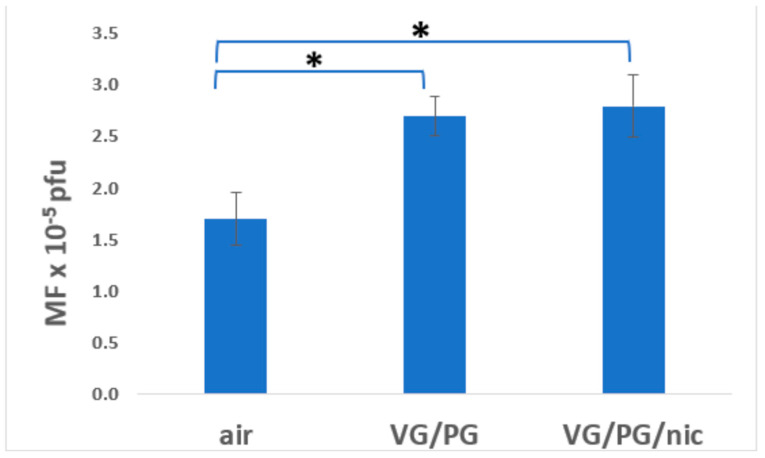
Effect of 3-month whole-body exposure of C57 *lacI* mice (Big Blue) to e-cig aerosols or filtered air on mutagenesis in bladder urothelial tissue. E-cig aerosol without nicotine (VG/PG); E-cig aerosol with nicotine, (VG/PG/nic), pfu (plaque-forming units). All groups consist of females + males. n = 13 for air, 10 for VG/PG, and 9 for VG/PG/nic groups. *, *p* < 0.02 vs. filtered air group in one-tailed *t*-test; error bars, mean ± SEM.

**Figure 4:**
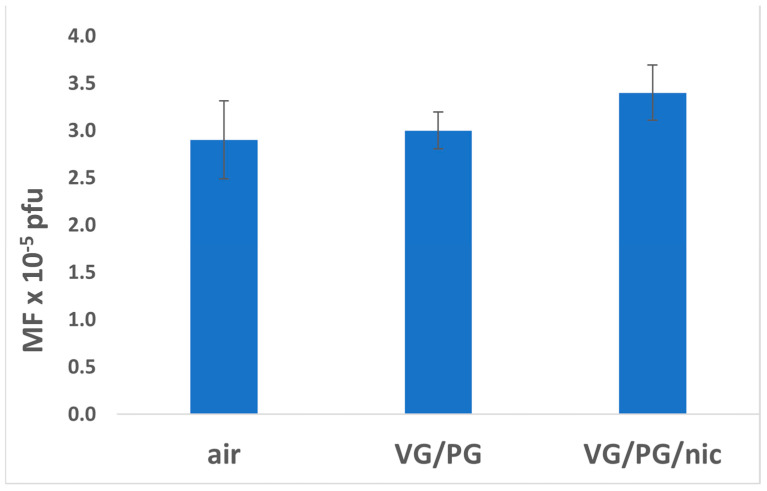
Effect of 3-month whole-body exposure of C57 *lacI* mice (Big Blue) to e-cig aerosols or filtered air on mutagenesis in liver. E-cig aerosol without nicotine (VG/PG); E-cig aerosol with nicotine, (VG/PG/nic), pfu (plaque-forming units). All groups consist of females + males. n = 13 for air, 10 for VG/PG, and 12 for VG/PG/nic groups. Error bars, mean ± SEM.

To more clearly illustrate the comparative mutagenesis in each exposure group (males + females), graphical representations are included (Figure 1, Figure 2, Figure 3 and Figure 4). These figures depict mutagenesis in the four tissues of interest after exposure to filtered air, VG/PG, or VG/PG + nicotine.

### 3.1. Lung

Mutagenesis in the mouse lung was increased (*p* ≤ 0.01) almost two-fold after exposure to the VG/PG/nicotine aerosol compared to either filtered air or VG/PG alone (Table 1, Figure 1). There were no significant differences in lung tissue mutagenesis between the sexes (Table 1).

### 3.2. Tongue

Mutagenesis in the mouse tongue was increased (*p* = 0.05) by about 15% in the VG/PG/nicotine group compared to the filtered air group (Table 1, Figure 2). Mutagenesis was significantly higher in males than in females in the VG/PG group (Table 1), but there were only four female mice, so the small group size may have led to a chance result. There were no significant differences in mutagenesis between males and females in any of the other treatment groups.

### 3.3. Bladder Urothelium

Mutagenesis in bladder urothelial tissue was significantly increased by about 60% in the VG/PG and the VG/PG/nicotine groups compared with the filtered air group (Figure 3, Table 1). The level of mutagenesis was similar between the VG/PG and VG/PG/nicotine groups. Mutagenesis in all three groups was greater in females than in males. This sex difference was statistically significant in the air and VG/PG/nicotine groups but not in the VG/PG group (Table 1).

### 3.4. Liver

Mutagenesis in liver tissue was similar in all three exposure groups. Although the differences failed to reach statistical significance, the level of mutagenesis was highest in the VG/PG/nicotine group (Figure 4, Table 1). Moreover, there were no significant differences in mutagenesis between any of the male and female treatment groups (Table 1).

## 4. Discussion

As conventional cigarettes are highly carcinogenic in the lung and a number of other organs, a concern exists concerning the relationship (if any) between long-term ENDS use and its potential for causing cancer. ENDSs usually contain a liquid mixture of VG, PG, flavorants, water, and often nicotine [18,19,20]. In addition to these components, when aerosolized, the mixture gives rise to a number of small aldehydes at levels similar to or greater than those in tobacco smoke [5,19,20]. However, e-cig aerosol does not contain most of the known carcinogens in tobacco smoke [21,22]. Hence, there is reason to believe that ENDS aerosols will prove less carcinogenic than tobacco smoke. However, there are multiple reports that ENDS aerosols damage DNA in cell cultures [23,24,25], and one publication reported that they cause lung cancer in mice [13]. As mutagenesis is often a driver of carcinogenesis [26], this study sought to investigate whether ENDS exposure in mice leads to mutagenesis.

We found that the exposure of mice to VG/PG/nicotine led to significantly enhanced mutagenesis relative to filtered air exposure in the lung, bladder, and tongue and a modest (albeit non-significant) enhancement in the liver. Nicotine can be metabolized to nornicotine and nitrosated to form NNN [6]. Nicotine may also be metabolized in vivo to the carcinogen NNK [6,7,27]. In support of this hypothesis is the detection of NNN in the saliva of vapers [7,27], NNAL in the urine of ENDS users [7,27], and the detection of the NNK-derived DNA adduct O^6^-methylguanine in tissues of E-cig aerosol-exposed mice [25].

In tissues other than the bladder, exposure to VG/PG alone did not increase the mutant fraction above that of the filtered air group. Sub-chronic exposure of mice to E-cig aerosols may result from licking the condensate from fur, mucociliary clearance/nasal clearance, inhalation after nasal passage, and swallowing, so that lung and upper aerodigestive tissues would be exposed. The bladder may be exposed to both unmetabolized E-cig aerosol components and their metabolites. The liver is exposed via circulation and likely experiences lower concentrations of E-cig aerosol components.

Previously, it was reported that about 20% of E-cig aerosol-exposed mice developed lung adenocarcinomas and bladder hyperplasia after a 54-week exposure [13]. Lung tumors were found in the nicotine-containing aerosol group but not in the VG/PG alone aerosol-exposed mice. Our finding of mutagenesis in the lungs of the VG/PG/nicotine aerosol-exposed group, and not in the VG/PG group, is consistent with this previous publication. In the same previous study, in the bladder, urothelial hyperplasia was observed in all of the VG/PG/nicotine-treated mice and a small percentage of VG/PG-treated mice. In this study, we observed mutagenesis in bladder urothelial DNA after a 3-month exposure in both the VG/PG/nicotine and VG/PG aerosol groups. Thus, mutagenesis induced by ENDS aerosols in the bladder could be the result of known ENDS-derived mutagens in urine and possibly additional agents that accumulate in urine, such as the aldehydes that form from oxidized VG and/or PG [28].

### Limitations of This Study and Future Research

A limitation of this study was the lack of a nicotine-only exposure group. Results from the use of such a control group could answer the question of whether nicotine alone is sufficient to induce mutagenesis or whether the aerosolized combination with VG/PG is necessary. Nicotine is generally not considered carcinogenic, and in current ENDSs, it is aerosolized with VG/PG, so current vapers do not have exposure simply to aerosolized nicotine. Additionally, the ENDS liquids used in the current study did not contain flavorants, and many ENDS liquids contain flavorants. There are hundreds of different flavorants and delivery systems available, and if any of these give rise to mutagenic products in the aerosol, users of those products would be subject to additional risk. Also, in many ENDS or cigarette inhalation experiments in rodents, the animals do not orally inhale directly from the device, but they breathe the atmosphere in the chamber and are also exposed through grooming, so there are exposure differences compared to humans. Finally, the device and the duration and intensity of exposure differ from person to person, and the exposure conditions in our study represent only one set of conditions.

Future studies will investigate whether the exposure of mice to aerosolized nicotine without VG/PG results in mutagenesis and the potential effects of longer exposure periods on mutagenesis.

## 5. Conclusions

Three months of exposure of *lacI* mice to nicotine-containing ENDS aerosol led to mutagenesis in the lung, bladder, and tongue, but not the liver. Exposure to ENDS aerosol without nicotine was only mutagenic in bladder urothelial tissue. These results support the hypothesis that exposure to ENDS aerosols represents a risk factor for carcinogenesis and mutagenesis. Additionally, this in vivo assay for mutagenesis is a useful intermediate-term assay for the detection of potential genotoxic effects associated with ENDS aerosols.

## Figures and Tables

**Table 1:** Effect of E-cig aerosols on mutagenesis in selected organs of *lacI* (BigBlue) mice.

Exposure	Target Organ	Sex	No. of Mice	Mutant Fraction ^1^	SD
air	**lung**	M	6	2.10	1.43
		F	7	3.05	0.99
		*M* + *F*	*13*	*2.62*	*1.20*
VG/PG		M	6	2.31	0.79
		F	4	2.56	1.54
		*M* + *F*	*10*	*2.41*	*1.0*
VG/PG/nicotine		M	6	4.79	1.93
		F	6	4.00	1.82
		*M* + *F*	*12*	*4.44*	*1.83*
air	**bladder**	M	6	1.39	0.81
		F	7	2.02	0.93
		*M* + *F*	*13*	*1.70*	*0.89*
VG/PG		M	6	2.32	0.27
		F	4	3.17	0.72
		*M* + *F*	*10*	*2.75*	*0.67*
VG/PG/nicotine		M	5	2.53	1.05
		F	4	3.09	1.17
		*M* + *F*	*9*	*2.78*	*1.07*
air	**tongue**	M	6	2.45	0.68
		F	7	2.92	0.29
		*M* + *F*	*13*	*2.68*	*0.61*
VG/PG		M	6	3.39	0.76
		F	4	1.93	0.31
		*M* + *F*	*10*	*2.81*	*0.91*
VG/PG/nicotine		M	6	3.33	0.62
		F	6	2.86	0.39
		*M* + *F*	*12*	*3.09*	*0.53*
air	**liver**	M	6	2.61	1.77
		F	7	3.90	1.11
		*M* + *F*	*13*	*2.89*	*1.38*
VG/PG		M	6	2.68	0.33
		F	4	3.54	0.78
		*M* + *F*	*10*	*3.03*	*0.64*
VG/PG/nicotine		M	6	3.86	0.69
		F	6	2.88	1.18
		*M* + *F*	*12*	*3.37*	*0.98*

^1^ Mean—in units of mutants/10^5^ plaque forming units; Italics indicate results for males + females.

## Data Availability

The data used to construct the table and figures may be found in the Supplementary Materials (Tables S1–S4).

## Supplementary Materials

The following supporting information can be downloaded at https://www.mdpi.com/article/10.3390/ijerph21121693/s1. Table S1. Mutant fractions in lungs of mice exposed to air, and E-cig aerosols with and without nicotine; Table S2. Mutant fractions in urothelial tissue of bladders of C57 *lacI* (BigBlue®)mice exposed to air, and E-cig aerosols with and without nicotine; Table S3. Mutant fractions in tongues of C57 *lacI* (BigBlue®) mice exposed to air and E-cig aerosols with and without nicotine; Table S4. Mutant fractions in liver of C57 *lacI* (BigBlue®) mice Exposed to air, and E-cig aerosols with and without nicotine.
