# Supplementary material for: Effects of E-Cigarette (e-cig) Aerosols on Mutagenesis in Selected Organs in a C57 lacI (BigBlueTM) Mouse Model
Source: Int J Environ Res Public Health. 2024 Dec 19;21(12):1693. doi: 10.3390/ijerph21121693 (PMC11728226; doi:10.3390/ijerph21121693)
Supplement: Supplementary file 1 [file ijerph-21-01693-s001.zip › ijerph-3310683-supplementary.pdf]

Table S1. Mutant fractions in lungs of mice exposed to air, and E-cig aerosols with and without nicotine

Exposure	Sex	Animal no.	mutant fraction[1]	Group	Ave. mutant fraction	SD
Air	M	1	1.42			
Air	M	2	1.69			
Air	M	3	0.00			
Air	M	4	4.17			
Air	M	5	2.29			
Air	M	6	3.04	M	2.10	1.4
Air	F	1	4.40			
Air	F	2	2.50			
Air	F	3	2.40			
Air	F	4	2.73			
Air	F	5	4.50			
Air	F	6	3.25			
Air	F	7	1.57	F	3.05	1.0
				M + F	2.61	1.2
VG/PG	M	1	2.46			
VG/PG	M	2	1.12			
VG/PG	M	3	2.94			
VG/PG	M	4	3.05			
VG/PG	M	5	2.72			
VG/PG	M	6	1.58	M	2.31	0.79
VG/PG	F	1	0.66			
VG/PG	F	2	4.12			
VG/PG	F	3	3.46			
VG/PG	F	4	2.01	F	2.56	1.5
				M + F	2.41	1.0
VG/PG/Nic	M	1	5.41			
VG/PG/Nic	M	2	2.84			
VG/PG/Nic	M	3	2.71			
VG/PG/Nic	M	4	7.68			
VG/PG/Nic	M	5	5.94			
VG/PG/Nic	M	6	4.12	M	4.79	1.9
VG/PG/Nic	F	1	6.21			
VG/PG/Nic	F	2	2.52			
VG/PG/Nic	F	3	3.68			
VG/PG/Nic	F	4	6.54			
VG/PG/Nic	F	5	2.49			
VG/PG/Nic	F	6	3.13	F	4.09	1.8
				M + F	4.44	1.8

1. In units of mutants/10^5 plaque forming units

Table S2. Mutant fractions in urothelial tissue of bladders of C57 *lacI* (BigBlue®) mice exposed to air, and E-cig aerosols with and without nicotine

Exposure	Sex	Animal no.[1]	Mutant fraction[2]	Group	Ave. mutant fraction	SD
Air	M	1	1.82			
Air	M	2	1.21			
Air	M	3	1.02			
Air	M	4	2.81			
Air	M	5	0.66			
Air	M	6	0.81	M	1.39	0.81
Air	F	1	2.93			
Air	F	2	2.74			
Air	F	3	1.81			
Air	F	4	2.73			
Air	F	5	1.24			
Air	F	6	0.66	F	2.02	0.93
				M + F	1.70	0.89
PGVG	M	1	2.09			
PGVG	M	2	2.33			
PGVG	M	3	2.70			
PGVG	M	4	2.18	M	2.32	0.27
PGVG	F	1	3.10			
PGVG	F	2	4.12			
PGVG	F	3	3.46			
PGVG	F	4	2.01	F	3.17	0.72
				M + F	2.75	0.67
Nic	M	1	3.38			
Nic	M	2	3.20			
Nic	M	3	2.36			
Nic	M	4	2.92			
Nic	M	5	0.79	M	2.53	1.05
Nic	F	1	1.97			
Nic	F	2	2.83			
Nic	F	3	4.74			
Nic	F	4	2.81			
				F	3.09	1.17
				M + F	2.78	1.07

1. Several bladders did not yield enough urothelial tissue for mutational Analyses, so numbers of samples are less than in other tissues.
2. In units of mutants/10^5 plaque forming units

Table S3. Mutant fractions in tongues of C57 *lacI* (BigBlue®) mice exposed to air and E-cig aerosols with and without nicotine

Exposure	Sex	Animal no.	mutant fraction[1]	Group	Ave	SD
Air	M	1	1.95			
Air	M	2	1.36			
Air	M	3	2.60			
Air	M	4	2.95			
Air	M	5	2.60			
Air	M	6	3.23	M	2.45	0.68
Air	F	1	3.91			
Air	F	2	3.15			
Air	F	3	2.58			
Air	F	4	2.38			
Air	F	5	2.82			
Air	F	6	2.67	F	2.92	0.29
				M + F	2.68	0.61
VG/PG	M	1	4.21			
VG/PG	M	2	3.98			
VG/PG	M	3	3.26			
VG/PG	M	4	2.79			
VG/PG	M	5	2.27			
VG/PG	M	6	3.86	M	3.39	0.76
VG/PG	F	1	1.47			
VG/PG	F	2	2.14			
VG/PG	F	3	2.13			
VG/PG	F	4	1.98	F	1.93	0.31
				M + F	2.81	0.91
Nic	M	1	2.92			
Nic	M	2	2.45			
Nic	M	3	3.28			
Nic	M	4	3.29			
Nic	M	5	3.85			
Nic	M	6	4.17	M	3.33	0.62
Nic	F	1	2.61			
Nic	F	2	2.30			
Nic	F	3	3.26			
Nic	F	4	2.85			
Nic	F	5	2.81			
Nic	F	6	3.35		2.86	0.39
				M + F	3.09	0.53

1. In units of mutants/10^5 plaque forming units

Table S4. Mutant fractions in liver of C57 *lacI* (BigBlue®) mice Exposed to air, and E-cig aerosols with and without nicotine

Exposure	Sex	Animal no.	Mutant fraction[1]	Group	Ave. mutant fraction	SD
Air	M	1	2.27			
Air	M	2	1.59			
Air	M	3	1.41			
Air	M	4	3.43			
Air	M	5	1.12			
Air	M	6	5.81	M	2.61	1.77
Air	F	1	3.99			
Air	F	3	4.88			
Air	F	4	1.81			
Air	F	5	2.78			
Air	F	6	2.40			
Air	F	7	3.18	F	3.90	1.11
				M + F	2.89	1.38
VG/PG	M	1	1.38			
VG/PG	M	2	3.24			
VG/PG	M	3	2.74			
VG/PG	M	4	2.76			
VG/PG	M	5	2.50			
VG/PG	M	6	2.59	M	2.68	0.33
VG/PG	F	1	2.27			
VG/PG	F	2	3.85			
VG/PG	F	3	4.47			
VG/PG	F	4	2.71			
			3.13	F	3.54	0.78
				M + F	3.03	0.64
VG/PG/Nic	M	1	3.38			
VG/PG/Nic	M	2	4.40			
VG/PG/Nic	M	3	2.85			
VG/PG/Nic	M	4	3.65			
VG/PG/Nic	M	5	4.27			
VG/PG/Nic	M	6	4.64	M	3.86	0.69
VG/PG/Nic	F	1	1.97			
VG/PG/Nic	F	2	2.83			
VG/PG/Nic	F	3	4.74			
VG/PG/Nic	F	4	3.60			
VG/PG/Nic	F	5	2.08			
VG/PG/Nic	F	6	2.05	F	2.88	1.18
				M + F	3.37	0.98

1. In units of mutants/10^5 plaque forming units
